# Depressive disorders: systematic review of approved psychiatric medications (2009-April 2025) and pipeline phase 3 medications

**DOI:** 10.1186/s12888-025-07141-3

**Published:** 2025-10-07

**Authors:** Waguih William IsHak, Drew Hirsch, Sabrina Renteria, Jayant Totlani, Nathalie Murphy, Tiffany Chang, Rasha Abdelsalam, Mohamed Salem, Ashley Meyer, Shirley Keerthana, Angela Liu, Lorena Contreras, Emile Tadros, Rebecca Hedrick, Itai Danovitch, Robert N. Pechnick

**Affiliations:** 1 Department of Psychiatry, Cedars-Sinai Medical Center, 8730 Alden Drive, Suite E-132, Los Angeles, CA 90048 USA; 2 Department of Psychiatry, University of California Los Angeles, David Geffen School of Medicine, Los Angeles, CA USA; 3 University of California Irvine School of Medicine, Irvine, CA USA; 4 Department of Pharmacology, Western University of Health Sciences College of Osteopathic Medicine of the Pacific, Pomona, CA USA

**Keywords:** Psychiatric medications, FDA-approved, Depressive disorders, Psychiatric disorders

## Abstract

**Background:**

Depressive disorders remain a significant public health concern, with substantial personal, social, and economic impacts worldwide. The purpose of this systematic review is to examine the psychiatric medications approved by the FDA from 2009 through early 2025, as well as investigational drugs in Phase 3 clinical trials for depressive disorders, with a focus on their mechanisms of action, indications, evidence for efficacy, dosing, and adverse effect profiles.

**Methods:**

We conducted a systematic search of the FDALabel Database for FDA-approved medications for depressive disorders, using the criteria: labeling type “Human Prescription Drug,” application type “NDA,” and “depression” or “major depressive disorder” under “Indications and Usage.” To identify medications in Phase 3 clinical trials or awaiting FDA approval, we searched the US Clinical Trials Registry with filters for condition “depression” or “major depressive disorder,” trial phase “Phase III,” and study dates from 01/01/2009 to 4/1/2025. Additional information on included drugs was obtained via a PubMed literature search.

**Results:**

From 2009 through early 2025, the FDA approved 15 medications for depressive disorders, and there are currently 18 pipeline medications in Phase 3 clinical trials. Notable advancements during this period include a large number of approved and Phase 3 antidepressants with mechanisms that deviate from the monoamine hypothesis, particularly those targeting glutamatergic NMDA receptors, GABA-A receptors, and kappa-opioid receptors. Moreover, several antidepressants (approved and in development) function as partial agonists at the 5-HT1A receptor, serotonin-norepinephrine reuptake inhibitors, and previously approved neuroleptics. Of note, every oral antidepressant approved by the FDA during this time is scheduled as once-daily.

**Conclusions:**

Our results show numerous FDA approvals of medications for depressive disorders. Phase 3 psychiatric medications for depressive disorders seem to show novel mechanisms of action, modes of administration, and side effects.

**Supplementary Information:**

The online version contains supplementary material available at 10.1186/s12888-025-07141-3.

## Introduction

Over the past few decades, there has been significant progress in the development of new medications for depressive disorders. The purpose of this systematic review is to examine recently approved (post-2009) and Phase 3 investigational medications for depressive disorders to comprehensively describe their mechanisms of action, indications, evidence of efficacy, dosing, practical implementation considerations, and adverse effects. Our goal is to provide prescribers with a pragmatic and informative review of recently approved psychiatric medications to assist clinicians in becoming familiar with newer agents, particularly those that might be underutilized.

Depressive disorders represent a significant public health burden, afflicting an estimated 350 million people worldwide [1]. In 2020, the prevalence of depression among adults in the US was 18.5%, per age-standardized data from the CDC’s 2020 Behavioral Risk Factor Surveillance System [2]. Despite currently approved pharmacological and non-pharmacological interventions, a significant proportion of individuals with depression continue to experience inadequate relief of symptoms. Treatment resistance, defined as the persistence of depressive symptoms despite adequate trials of two or more antidepressant medications, represents a major clinical challenge. It is estimated that at least 30% of individuals with depression exhibit resistance to conventional antidepressant therapies [3], and as such, there is a pressing need for both the development of novel antidepressants and their subsequent uptake into clinical practice.

## Methods

To identify FDA-approved medications for depressive disorders, we conducted a systematic search of the FDALabel Database. The search was performed using the following criteria: (1) labeling types restricted to “Human Prescription Drug” to include only drugs regulated for human use and exclude those approved for over-the-counter use; (2) application type limited to “NDA"(New Drug Applications) to exclude authorized generic fornulations from the search and maintain relevancy; (3) labeling section(s) containing the terms"depression OR major depressive disorder” within the “Indications and Usage” category; and (4) market status list as “active” between January 1, 2009 and April 1, 2025. We manually screened drug labels to exclude medications approved for conditions other than unipolar depression.

To identify pipeline medications currently in Phase 3 clinical trials, as well as those that have completed Phase 3 and are awaiting approval from the FDA, we performed a comprehensive search of the US Clinical Trials Registry (ClinicalTrials.gov). The search was conducted using for the following criteria: (1) condition or disease limited to"depression"or"major depressive disorder"to maintain specificity to unipolar depression; (2) trial phase restricted to “Phase III” to include only those medications in the later stage of clinical development with results of confirmatory trials; and (3) study start from January 1, 2009 to April 1, 2025, to maintain a focus on recently completed and ongoing trials. We applied additional criteria to exclude trials that were suspended, withdrawn, or terminated during this period, as these trials are unlikely to yield an FDA-approved medication. Furthermore, trials with less than 50 participants in order to exclude pilot studies based on the statistical literature [4]. We also excluded studies that tested a non-drug or over-the-counter intervention, or those aiming to treat a condition other than unipolar depression, were excluded. To identify only compounds remaining in the later stages of development and maintain relevancy, compounds that failed to meet their primary endpoint in all RCTs or were announced to no longer be in active development were also excluded.

Both sources were last searched on April 2nd, 2025. All identified approved and pipeline medications were independently reviewed and assessed by two reviewers. Any discrepancies were resolved through discussion to reach a consensus on the final list of medications included in this systematic review. After identifying medications that fulfilled the selection criteria, additional information on the included drugs was gathered through a PubMed literature search.

It is important to note the potential risk of bias in the selection of evidence, both within individual studies and across studies, including publication bias. For example, results from negative or non-significant results from clinical trials may remain unpublished. The reporting of this systematic review was conducted in accordance with the standards of the Preferred Reporting Items for Systematic Review and Meta-Analysis (PRISMA) Statement [5].

## Results

**Fig. 1:**
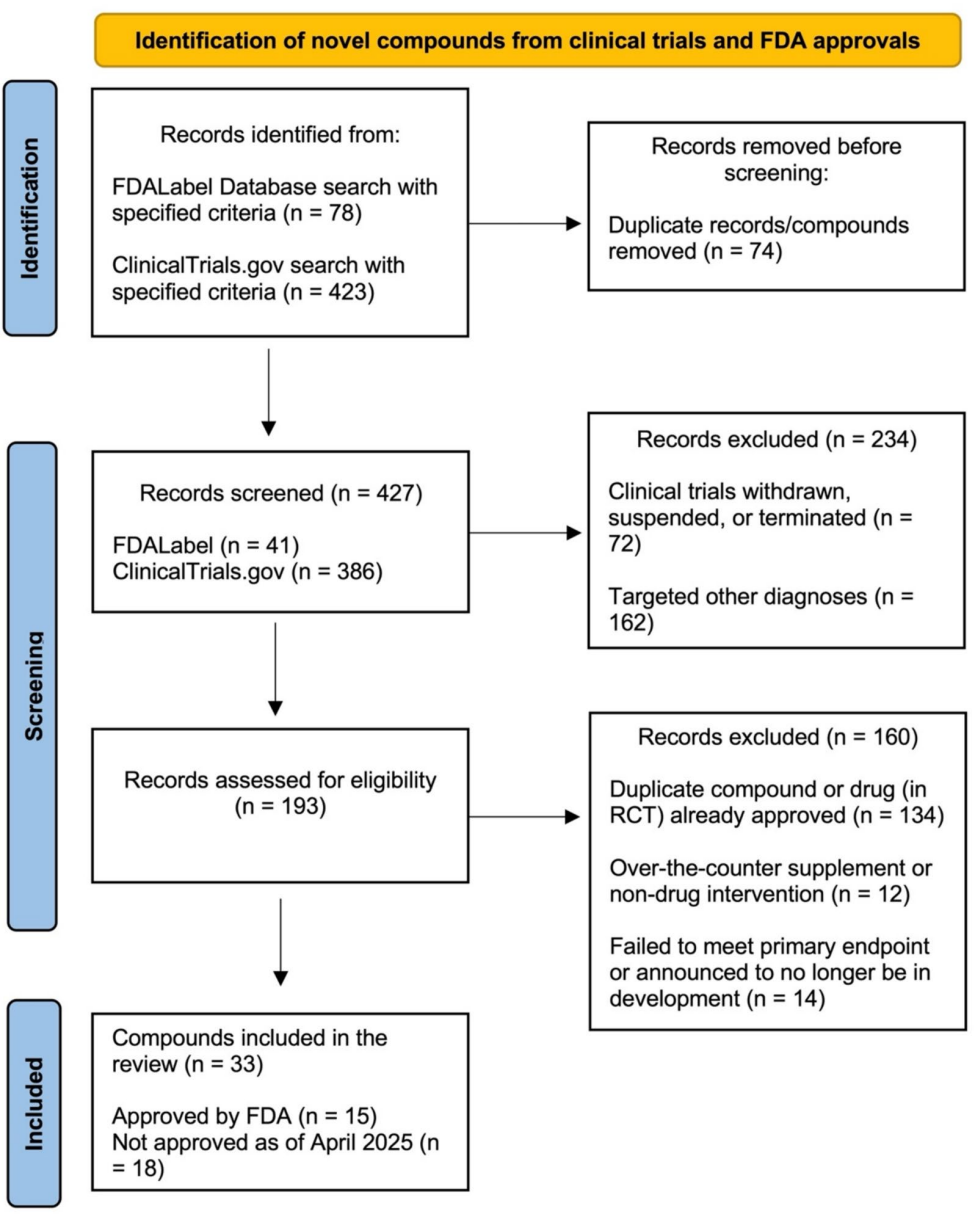
PRISMA flow diagram of included novel antidepressants from clinical trials and FDA approval data

From 2009 through early 2025, we found that the Food and Drug Administration (FDA) approved 15 medications for depressive disorders (Table 1), and 18 pipeline medications are currently in Phase 3 clinical trials (Table 2.). A flow diagram outlining the identification, classification, and organization of these medications is presented in Fig. 1. FDA-approved medications in Table 1 are organized alphabetically by the generic name of each medication and include each medication’s trade name and date of approval by the FDA. Medications currently in Phase 3 with results of confirmatory trials in Table 2. are organized alphabetically by their generic names and include other names (such as developmental code names), as well as the year each drug entered Phase 3 trials. For each psychiatric medication, detailed descriptions are provided below, including the medication class, mechanisms of action, indications for labeled and off-label uses, evidence for efficacy, practical implementation issues (cost, special procedures, or restrictions), and reported adverse effects.

**Table 1:** Approved medications for depressive disorders from 2009 through April 2025

Generic name	Trade name	Approval year
Primary Agents for Major Depression		
1. Bupropion hydrochloride extended-release	Forfivo XL	2011
2. Duloxetine delayed-release	Drizalma Sprinkle	2019
3. Gepirone	Exxua	2023
4. Levomilnacipran	Fetzima	2011
5. Trazodone extended-release	Oleptro	2010
6. Vilazodone	Viibryd	2011
7. Vortioxetine	Trintellix	2013
Augmenting Agents for Major Depression		
1. Aripiprazole oral film	Opipza	2024
2. Brexpiprazole	Rexulti	2015
3. Cariprazine	Vraylar	2022
4. Quetiapine extended-release	Seroquel XR	2009
Combination Therapy for Major Depression		
1. Dextromethorphan-bupropion	Auvelity	2023
Augmenting Agents for Treatment-Resistant Depression		
1. Esketamine nasal spray	Spravato	2020
Primary Agents for Postpartum Depression		
1. Brexanolone	Zulresso	2019
2. Zuranolone	Zurzuvae	2023

**Table 2:** Medications for Depressive Disorders in the Pipeline (Phase-3) as of April 1, 2025

**Generic Name**	**Trade Name**	**Phase-3 Year**
**Primary Agents for Major Depression**		
1. Ammoxetine	(S)-071031B	2025
2. Navacaprant	BTRX-335140, NMRA-140	2023
3. Solriamfetol	Sunosi, SKL-N05	2024
**Augmenting Agents for Major Depression**		
1. Aticaprant	JNJ-67953964, CERC-501	2011
2. Buprenorphine	Subutex, Belbuca	2021
3. Celecoxib	Celebrex, Onsenal, Elyxyb	2020
4. Deuterated Psilocybin Analog	CYB003	2024
5. Esmethadone	REL-1017	2021
6. Lumateperone	Caplyta	2020
7. Milsaperidone	VHX-896, P-88	2025
8. Osavampator	NBI-1065845, TAK-653	2025
9. Pramipexole	Mirapex, Mirapex ER	2022
10. Roflumilast	Daliresp	2025
11. Seltorexant	MIN-202, JNJ-42847922	2020
12. Ulotaront	SEP-363856; SEP-856	2023
**Primary Agents for Treatment-Resistant Depression**		
1. Psilocybin	COMP360	2022
**Adjunctive Agents for Treatment-Resistant Depression**		
1. Ketamine (racemic)	Ketalar	2015
2. Minocycline	Minocin, Amzeeq	2020

### Detailed descriptions of approved medications for depressive disorders from january 1, 2009, through april 1, 2025 (summarized in Table 3)

**Table 3:** Summary of approved agents for depressive disorders from January 1, 2009, through April 1, 2025

Name, year approved	Mechanism of action	Route and dose	Notes to clinicians including effects on sedation, weight/lipids, sexual dysfunction and QTc
Primary Agents for Major Depression			
1. Bupropion hydrochloride extended-release (Forfivo XL), 2011	Noradrenergic and dopaminergic reuptake inhibitor	Oral 450 mg in the morning	This form contains 450 mg in one extended-release tablet. Side effects include insomnia, weight loss, no sexual dysfunction, and no effects on QTc
2. Duloxetine (Drizalma Sprinkle), 2019	Serotonin -norepinephrine reuptake inhibitor	Oral 30–60 mg daily	Sedation, nausea, and constipation
3. Gepirone Extended-Release (Exxua), 2023	Partial 5-HT1A receptor agonist	Oral 18.2–72.6 mg daily	QTc prolongation, and weight gain
4. Levomilnacipran (Fetzima), 2009	Serotonin -norepinephrine reuptake inhibitor	Oral 40 mg-120 mg daily	Monitor for BP elevation Dose-related effects included sexual side effects and urination difficulty
5. Trazodone ER (Oleptro), 2010	5-HT2A and 5-HT2B receptor antagonist, -HT1A receptor partial agonist and reuptake inhibitor	Oral 150–375 mg daily	Daytime sedation, QTc prolongation, sexual dysfunction, and priapism (which was not reported in initial studies not powered to detect it)
6. Vilazodone (Viibryd), 2011	5-HT reuptake inhibitor and 5-HT1A receptor partial agonist	Oral 20–40 mg daily with food	Very low frequency of sexual side effects
7. Vortioxetine (Trintellix), 2013	Nonselective serotonin reuptake inhibitor; 5-HT1A receptor agonist, 5-HT1B receptor partial agonist; 5-HT3 and 5-HT7 receptor antagonist. *	Oral 5–20 mg daily	Very low frequency of sexual side effects
Augmenting Agents for Major Depression:			
1. Aripiprazole oral film (Opipza), 2024	D2 and 5-HT1A partial agonist, and 5-HT2A receptor antagonist	Oral film 2–15 mg daily	Notable adverse effects include akathisia, restlessness, insomnia, constipation, fatigue, and blurred vision
2. Brexpiprazole (Rexulti), 2015	D2 and 5-HT1A partial agonist, and 5-HT2A receptor antagonist	Oral 1–3 mg daily	Akathisia, somnolence, and weight gain
3. Cariprazine (Vraylar), 2022	Partial agonist at D2, D3, and 5-HT1A receptors, and antagonist at 5-HT2A receptors	Oral 1.5–4.5 mg/day	Akathisia, insomnia
4. Quetiapine Extended-Release (Seroquel XR), 2009	5-HT2A and D2 receptor antagonist	Oral 50–300 mg daily	Daytime sedation, weight gain, dyslipidemia
Combination Therapy for Major Depression			
1. Dextromethorphan + bupropion (Auvelity), 2023	Dextromethorphan prodrug for dextrorphan) is an NDMA receptor antagonist and non-selective serotonin reuptake inhibitor,* bupropion is noradrenergic and dopaminergic reuptake inhibitor	Oral 45 mg dextromethorphan + 105 mg bupropion once daily then increase to twice daily after 3 days	Somnolence, sexual dysfunction
Augmenting Agents for Treatment-Resistant Depression			
1. Esketamine nasal spray (Spravato), 2020 for treatment refractory depression	NMDA receptor antagonist *	Intranasal 56 mg-84 mg 1–2 times weekly	Needs office monitoring for BP elevation, dissociation. Schedule III drug
Primary Agents for Postpartum Depression			
1. Brexanolone (Zulresso), 2019	GABA-A receptor modulator *	Intravenous infusion (continuous) 60–90 µg per kilogram per hour for 60 h	Headache, dizziness, and somnolence. Schedule IV drug. Only available through Brexanolone REMS
2. Zuranolone (Zurzuvae), 2023	GABA receptor modulator *	Oral 30–50 mg nightly	Headache, dizziness, and somnolence. Schedule IV drug. Easier administration than brexanolone

*Abbreviations*: *D2, D3, D4* Dopamine receptors, *5HT* Serotonin receptors, *H* Histamine receptors, *GABA* Gamma Amino Butyric Acid receptors, *NMDA* N-methyl-D-aspartate receptor, *IM* Intramuscular, *IN* Intranasal, *IV* Intravenous, *SC* subcutaneous

^*^Novel mechanism of action

#### Primary agents for major depression:

1. Bupropion hydrochloride extended-release (Forfivo XL)

*Overview*: Forfivo XL is a high-strength (450 mg) daily form of bupropion [6]. It was approved by the FDA in 2011, and it acts as a dopamine and norepinephrine reuptake inhibitor [7].

*Indications*: MDD (2011).

*Dose and Route*: Oral tablet. Initial dose: 150 mg once daily in the morning; can increase to 300 mg once daily after 4 days; maximum single dose: 450 mg once daily.

*Evidence*: In a four-week placebo-controlled trial, patients were randomized to receive either bupropion 450 mg/day (*n* = 34), bupropion 300 mg/day (*n* = 36), or placebo (*n* = 39). At the endpoint, the least squares (LS) mean change from baseline in HDRS score was −17.4 (SE 1.7) for the bupropion 450 mg/day group, −15.5 (SE = 1.7) for the bupropion 300 mg/day group, and −11.5 (SE 1.6) for the placebo group [6, 8]. Additionally, a 2016 systematic review and meta-analysis found that monotherapy with bupropion was effective in reducing scores of depression in five of the six identified double-blinded, placebo-controlled RCTs. Of all identified trials evaluating bupropion in the treatment of MDD, including trials comparing bupropion to other antidepressants, bupropion was positively effective in their primary outcome measure in 24 of 27 trials [7].

*Practical Issues and Implementation*: Given once daily in the morning. Contraindicated in a seizure disorder, current or prior diagnosis of bulimia or anorexia nervosa, abrupt discontinuation of alcohol, benzodiazepines, barbiturates, antiepileptic drugs, and in patients taking monoamine oxidase inhibitors (MAOIs).

*Adverse Effects*: Irritability, loss of appetite, insomnia.

2. Duloxetine delayed-release (Drizalma Sprinkle)

*Overview*: Duloxetine (Drizalma Sprinkle) is a delayed-release oral capsule formulation of duloxetine that can be swallowed whole, opened and sprinkled over food, or administered via nasogastric tube, making it easy for patients who have trouble swallowing [9].

*Indications*: MDD in adults (2019), GAD in adults and pediatric populations (7–17 years) (2019), adult diabetic peripheral neuropathy, chronic musculoskeletal pain (2019), and fibromyalgia (2021).

*Dose and Route*: Capsule form. 30 to 60 mg daily. For depression, the initial 40 mg/day in 2 doses; can increase to 60 mg/day in 1–2 doses if necessary; maximum dose generally 120 mg/day.

*Evidence*: In four placebo-controlled RCTs, duloxetine delayed-release capsules showed efficacy in adult patients with MDD. In studies 1 (*n* = 123) and 2 (*n* = 128), 60 mg/day of duloxetine for 9 weeks reduced LS mean HAM-D-17 scores from baseline by 10.9 (SE = 0.7; place) and 10.5 (SE = 2.2), relative to placebo (*n* = 122 and *n* = 139, respectively) at 6.1 (SE = 0.69) and 8.3 (SE = 8.3), respectively. In study 3, patients were randomized to 20 (*n* = 86) or 40 (*n* = 91) mg twice daily or placebo (*n* = 89) for 8 weeks. Both doses showed efficacy over placebo, reducing LS mean HAM-D-17 scores from baseline by 7.4 (SE = 0.8) and 8.6 (SE = 8.6), respectively, relative to placebo (5.0, SE = 0.81). Lastly, in study 4, patients were randomized to 40 (*n* = 95) or 60 (*n* = 93) mg twice daily or placebo (*n* = 93). Both 40 mg and 60 mg BID produced significant LS mean changes from baseline over 8 weeks of 11.0 (SE = 0.49) and 12.1 (SE = 12.1), respectively, relative to placebo (8.8, SE = 0.5) [9].

*Practical Issues and Implementation*: Helpful for patients with swallowing difficulties in or out of long-term facilities or hospitals.

*Adverse Effects*: Nausea, dry mouth, and headache.

3. Gepirone (EXXUA)

*Overview*: Gepirone is an orally administered antidepressant that acts as a serotonin 5-HT1A receptor agonist [10].

*Indications*: MDD (2023).

*Dose and Route*: Oral 18.2 to 72.6 mg once per day.

*Evidence*: In two placebo-controlled 8-week RCTs, 18.2–72.6 mg/day extended-release gepirone produced statistically significant improvements in HAM-D-17 scores relative to placebo through weeks 3–8. In study 1, gepirone produced an LS mean change from baseline (CFB) of 9.04 (*n* = 101; SE = 0.78), whereas placebo produced a change of 6.75 (*n* = 103; SE = 0.78). In study 2, gepirone produced an LS mean CFB of 10.22 (*n* = 116; SE = 0.75), whereas placebo produced a change of 7.96 (*n* = 122; SE = 0.73) [10]. Furthermore, administration of extended-release gepirone in patients with MDD (*n* = 126) showed significant improvement in HAM-D-17 scores and was significantly more effective in preventing relapse of depressive symptoms compared to placebo (*n* = 124) [11, 12].

*Practical Issues and Implementation*: Taken once daily with food. Gepirone should be titrated up from the starting dose (18.2 mg), and depending on clinical response and tolerability, may be increased to 36.3 mg by day 4, 54.5 mg after day 7, and 72.6 mg after an additional week.

*Adverse Effects*: Commonly reported adverse effects include nausea, dizziness, vertigo, headache, and insomnia.

4. Levomilnacipran extended-release (Fetzima)

*Overview*: Levomilnacipran (Fetzima) is an orally administered antidepressant indicated for the treatment of MDD in adults [13]. It is closely related to milnacipran, which is approved for fibromyalgia but not MDD. Levomilnacipran is a serotonin and norepinephrine reuptake inhibitor that has a similar tolerability and efficacy profile to other drugs in the class [14].

*Indications*: MDD (2013).

*Dose and Route*: Extended-release Capsule. Initial dose 20 mg once daily for 2 days, then increase to 40 mg once daily; can increase by 40 mg/day every 2 or more days; maximum recommended dose 120 mg once daily.

*Evidence*: A Phase 3, randomized, double-blind, placebo-controlled study evaluated the efficacy of levomilnacipran sustained release (SR) in patients with major depressive disorder (MDD) using the MADRS as the primary outcome measure. The study included 724 patients who were randomly assigned to receive either placebo (*n* = 179) or levomilnacipran SR at doses of 40 mg (*n* = 181), 80 mg (*n* = 181), or 120 mg (*n* = 183) once daily for 8 weeks. By the endpoint, the least squares mean difference (LSMD) for change from baseline in MADRS total score was −3.23 for the 40 mg dose (*p* = 0.0186), −3.99 for the 80 mg dose (*p* = 0.0038), and −4.86 for the 120 mg dose (*p* = 0.0005) [14].

*Practical Issues and Implementation*: Regular monitoring of blood pressure and heart rate is recommended due to the potential for hypertension and tachycardia. Additionally, gradual dose reduction is advised when discontinuing the medication to minimize withdrawal symptoms.

*Adverse Effects*: Levomilnacipran was generally safe and well tolerated. Common side effects were dizziness, nausea, tachycardia, palpitations, hypertension, and hypotension.

5. Trazodone ER (Oleptro)

*Overview*: Trazodone (Oleptro) is an orally administered drug and has an antagonist effect on alpha-1 adrenergic receptors and H1 histamine receptors in low doses, and at increased doses, it inhibits the reuptake of 5HT [15]. It also acts as a partial agonist at 5HT1A receptors.

*Indications*: Major depression, insomnia, and GAD.

*Dose and Route*: Tablet. Initial dose: 150 mg/day in divided doses; can increase every 3–4 days by 50 mg/day as needed; maximum 400 mg/day (outpatient) or 600 mg/day (inpatient), split into 2 daily doses.

*Evidence*: A Phase 3, placebo-controlled RCT assessing the efficacy, safety, and clinical benefit of once daily trazodone in the treatment of unipolar MDD was conducted with the change in HAMD-18 total score from baseline to week 8 as the primary outcome measure. Patients randomized to the trazodone group received a starting dose of 150 mg titrated up by 75 mg every 3–4 days to an optimal dose, with a maximum daily dose of 375 mg. By week 8, the least squares mean difference (LSMD) for change from baseline in MADRS total score was −11.4 (SD = 8.20) for the trazodone group (*n* = 202, *p* = 0.012) and −9.3 (SD = 7.94) for the placebo group (*n* = 204) [16].

*Practical issues and Implementation*: Trazodone has been associated with possible QTc interval prolongation [17].

*Adverse effects*: Adverse effects include urticaria, chills, flushing, priapism, and hypotension, among others.

6. Vilazodone (Viibryd)

*Overview*: Vilazodone (Viibryd) is an orally administered antidepressant that is a selective serotonin reuptake inhibitor as well as a 5HT1 receptor partial agonist [18]. It has been found to be efficacious in the treatment of MDD and GAD in adults [19–21].

*Indications*: MDD (2011), GAD (off-label).

*Dose and Route*: Immediate release tablet. Initial 10 mg/day; increase to 20 mg/day after one week; can increase to 40 mg/day after one more week; should be taken with food.

*Evidence*: In a double-blind placebo-controlled RCT, 481 adults with MDD were randomized to receive vilazodone (titrated to 40 mg/day) or placebo over 8 weeks [22]. The primary efficacy endpoint was the change in MADRS total score from baseline to the end of treatment. Vilazodone-treated patients (*n* = 231) showed a significantly greater improvement in MADRS scores compared to placebo (*n* = 232) (least-squares mean changes: −13.3 vs. −10.8, *p* = 0.009). Furthermore, a second study by Croft et al. (2014) evaluated vilazodone 40 mg/day over 8 weeks in 505 outpatients with MDD. The primary outcome was the change in MADRS total score from baseline to week 8. Vilazodone (*n* = 253) demonstrated a statistically significant improvement over placebo (*n* = 252), with a least squares mean difference in MADRS total score of −5.117 (95% CI: −6.886 to −3.347, *p* < 0.00001) [23].

*Practical Issues and Implementation*: Taken with meals.

*Adverse Effects*: Diarrhea, nausea, vomiting, and insomnia.

7. Vortioxetine (Trintellix)

*Overview*: Vortioxetine is an orally administered serotonin reuptake inhibitor, as a partial agonist of the 5-HT1B receptors, an agonist of 5-HT1A receptors, and an antagonist of the 5-HT3, 5-HT1D, and 5-HT7 receptors. It is FDA-approved for MDD and is also used off-label for anxiety [24]. It was first released as Brintellix and subsequently renamed to Trintellix to avoid confusion with Brilinta (ticagrelor).

*Indications*: MDD in adults (2013), GAD (off-label).

*Dose and Route*: Immediate release tablet. Oral 5 to 20 mg daily.

Initial 10 mg once daily; can decrease to 5 mg once daily or increase to 20 mg once daily, depending on patient response; maximum recommended dose is generally 20 mg once daily.

*Evidence*: A Phase 3, placebo-controlled, double-blinded clinical trial evaluating the efficacy of vortioxetine in depression included 462 subjects with major depressive disorder (MDD) who were randomized to receive either vortioxetine 10 mg (*n* = 155), vortioxetine 20 mg (*n* = 150), or placebo (*n* = 157) over 8 weeks. The primary outcome was the change in MADRS total score from baseline. By week 8, the mean reductions in MADRS total were −10.77 (SE = 0.807) for placebo, −12.96 (SE = 0.832) for vortioxetine 10 mg (*P* = 0.058 vs placebo), and −14.41 (SE = 0.845) for vortioxetine 20 mg (*P* = 0.002 vs placebo). Thus, unlike the 10 mg dose, vortioxetine 20 mg demonstrated a statistically significant improvement in MADRS total score compared to placebo. Additionally, the response rates (≥ 50% decrease from baseline in MADRS total score) were 28.4% for placebo, 33.8% for vortioxetine 10 mg, and 39.2% for vortioxetine 20 mg, with only the 20 mg dose showing a significant difference from placebo (*P* = 0.044) [25].

*Practical Issues and Implementation*: The maximum recommended dose is 10 mg for poor CYP2D6 metabolizers.

*Adverse Effects*: Common treatment-emergent adverse events in comparison to placebo were nausea, vomiting, headache, dizziness, and dry mouth [26, 27].

#### Augmenting agents for major depression:

1. Aripiprazole oral film (Opipza) (as adjunctive therapy)

*Overview*: Aripiprazole oral film (Opipza) is an atypical antipsychotic approved by the FDA in 2024 for the adjunctive treatment of major depressive disorder (MDD) in adults, among other indications. As a partial agonist of the dopamine D2 and 5-HT1A receptors, aripiprazole maintains a favorable side-effect profile relative to atypical antipsychotics [28].

*Indications*: Schizophrenia in patients ages 13 years and older (2002), as an adjunct for major depression in adults (2007), irritability associated with autism in pediatric patients 6 years and older (2009), Tourette’s disorder in pediatric patients 6 years of age and older (2014). As an oral film, aripiprazole was approved in 2024 for these indications [29].

*Dose and Route*: For adjunctive treatment of MDD, start at 2–5 mg/day; can increase to a recommended dosage of 5–10 mg/day, with a maximum dosage of 15 mg/day.

*Evidence*: No trials were found investigating the efficacy of the oral film formulation of aripiprazole in depressive disorders; however, there is considerable evidence for the adjunctive use of aripiprazole to treat depression. A meta-analysis by Spielmans et al. (2013) investigating the efficacy of adjunctive atypical antipsychotic treatment for MDD found that adjunctive aripiprazole had a statistically significant effect on remission rates with an odds ratio (OR) of 2.01 (95% confidence interval [CI], 1.48–2.73) and a number needed to treat (NNT) of 9. Additionally, aripiprazole showed significant effects on response rates with an OR of 2.07 (95% CI, 1.58–2.72) and an NNT of 7. The effect size for clinician-rated depression severity measures was reported as Hedges'g = 0.37 for MADRS [30].

*Practical Issues and Implementation*: Dissolve on top of the tongue once daily with or without food. Oral film preparations may be ideal for patients with dysphagia, who may have difficulty swallowing conventional tablets.

*Adverse Effects*: Akathisia, restlessness, insomnia, constipation, fatigue, and blurred vision.

2. Brexpiprazole (Rexulti) (as adjunctive therapy)

*Overview*: Brexpiprazole (Rexulti) is an orally administered partial agonist at serotonin 5-HT1A and dopamine D2 receptors with antagonist activity at serotonin 5-HT2A receptors [31]. It was approved to treat schizophrenia, as an adjunct in major depression, and for agitation in patients with dementia.

*Indications*: As an adjunct to antidepressants in MDD (2015), schizophrenia (2015), and for agitation in dementia (May 2023) as a standing medication (not PRN).

*Dose and Route*: The tablet is taken orally.

Initial 0.5–1 mg once daily; increase in weekly intervals up to 1 mg once daily and then up to 2 mg once daily; maximum dose 3 mg once daily.

*Evidence*: A meta-analysis by Kishi et al. (2019) investigated the efficacy of adjunctive brexpiprazole in MDD following treatment failure with at least one antidepressant. The analysis included nine double-blind, placebo-controlled RCTs (*N* = 3,391) and found that response rate with brexpiprazole showed a significant improvement compared to placebo (risk ratio [RR] = 0.93, 95% confidence interval [CI] = 0.89–0.97, number needed to treat [NNT] = 17). The remission rate also favored brexpiprazole (RR = 0.95, 95% CI = 0.93–0.98, NNT = 25). Secondary outcomes included MADRS score, which showed a standardized mean difference (SMD) of −0.20 (95% CI = −0.29, −0.11) [32].

*Practical Issues and Implementation*: Very similar to aripiprazole in pharmacodynamic profile without adverse effects on cognition [33, 34].

*Adverse Effects*: Akathisia, somnolence, headache, weight gain, and tremor [35].

3. Cariprazine (Vraylar) (as adjunctive therapy)

*Overview*: Cariprazine is an orally administered partial agonist at dopamine D2 and serotonin 5-HT1A receptors and antagonist activity at serotonin 5-HT2A receptors. It also acts on NMDA glutamatergic function in the prefrontal cortex [36]. It is already approved for the treatment of schizophrenia (2015), depressive episodes associated with bipolar I disorder (bipolar depression) (2017), and acute treatment of manic or mixed episodes associated with bipolar I disorder (2019). It is also approved as an adjunctive therapy for MDD (2022).

*Indications*: Adjunctive therapy to antidepressants for MDD (2022).

*Dose and Route*: Capsule form. 1.5–4.5 mg/day.

*Evidence*: A meta-analysis by Gill et al. (2024) included four RCTs and found that cariprazine augmentation in the treatment of MDD resulted in a moderate antidepressant effect with a standardized mean difference (SMD) in MADRS scores of −1.79 (95% CI: −2.89 to −0.69). The pooled response rate ratio (RR) was 1.21 (95% CI: 1.05 to 1.39, *P* = 0.008), indicating a significant improvement in response rates [36].

*Practical Issues and Implementation*: Significant reports of adverse effects in the majority of participants (79%) [37].

*Adverse Effects*: Common adverse effects reported during administration were nausea, restlessness, dizziness, and sedation [38].

4. Quetiapine extended-release (Seroquel)

*Overview*: Quetiapine (Seroquel) is an orally administered drug and, as an atypical antipsychotic, it is an antagonist of dopamine D2 and serotonin 5-HT2A receptors. It is approved as an adjunct for MDD [39].

*Indications*: Adjunctive treatment for MDD patients who have had an inadequate response with antidepressants alone (2009), treatment of bipolar depression and mania (2008), treatment of schizophrenia (2007).

*Dose and Route*: Oral tablet. 50 to 300 mg daily.

*Evidence*: A meta-analysis by Spielmans et al. evaluated the efficacy of adjunctive atypical antipsychotics in the treatment of major depressive disorder. The results demonstrated that adjunctive quetiapine significantly improved remission rates with an odds ratio (OR) of 1.79 (95% CI, 1.33–2.42) and a number needed to treat (NNT) of 9. Additionally, quetiapine showed significant effects on response rates with an OR of 1.53 (95% CI, 1.17–2.0) and an NNT of 10 [30].

*Practical issues and Implementation*: Increased mortality risk in elderly patients with dementia-related psychosis.

*Adverse effects*: Rapid adverse effects are pruritus, QT prolongation, hypotension, and sinus tachycardia. Early adverse effects are dysgeusia, maculopapular rash, increased urinary frequency, hiccups, hyperventilation, palpitations, and AV blocks. Delayed adverse effects are weight gain, seborrhea, epistaxis, ecchymosis, tinnitus, orthostatic hypotension, heart failure, and hearing loss.

#### Combination therapy for major depression:

1. Dextromethorphan-bupropion (Auvelity)

*Overview*: Auvelity is an orally administered drug that combines dextromethorphan, a prodrug of dextrorphan of an NMDA receptor antagonist, with bupropion, a dopamine-norepinephrine reuptake inhibitor [40]. Dextromethorphan is FDA-approved as a cough suppressant, and bupropion is FDA-approved as an antidepressant. AXS-05 received FDA Breakthrough Therapy designation for the treatment of MDD in 2019 and agitation in Alzheimer’s Disease in 2020.

*Indications*: MDD (2023).

*Dose and Route*: Capsule form. Oral 45 mg dextromethorphan + 105 mg bupropion once daily, then increase to twice daily after 3 days.

*Evidence*: In a Phase 3 placebo-controlled RCT of adults with MDD, dextromethorphan plus bupropion (*n* = 163) showed statistically significant superiority over placebo (*n* = 164) in attenuating symptoms of depression starting one week after initiation. By week six, dextromethorphan plus bupropion produced a least-squares mean change (LS MD) in MADRS total score of −15.9 points, compared to −12.0 points in the placebo group [41].

*Practical Issues and Implementation*: As a CYP2D6 inhibitor, bupropion prolongs the rapid metabolism of dextromethorphan, blocking it from being demethylated into dextrorphan, a dissociative.

*Adverse Effects*: Nausea, dizziness, dry mouth, decreased appetite, anxiety [41, 42].

#### Augmenting agents for treatment-resistant depression:

1. Esketamine nasal spray (Spravato)

*Overview*: Esketamine (Spravato) is an NMDA-antagonist nasal spray that is a Schedule III medication approved for severe forms of depression [43].

*Indications*: As adjunctive therapy in TRD in adults (2020), and depressive symptoms in adults with MDD with acute suicidal ideation or behavior (2020).

*Dose and Route*: 56 mg dose kit: unit-dose carton containing two 28 mg nasal spray devices.

Eighty-four mg dose kit: unit-dose carton containing three 28 mg nasal spray devices.

Induction phase (weeks 1–4): administer twice per week; day 1 starting dose 56 mg; subsequent doses 56 mg or 84 mg.

Maintenance phase (weeks 5–8): administer once weekly; 56 mg or 84 mg.Maintenance phase (weeks 9 and after): administer every 2 weeks or once weekly (use least frequent dosing to maintain remission/response); 56 mg or 84 mg.

*Evidence*: In a Phase 3 placebo-controlled RCT of adults with treatment-resistant depression newly initiated on a daily oral antidepressant (duloxetine, escitalopram, sertraline, or venlafaxine), twice weekly 56 and 84 mg doses of intranasal esketamine (*n* = 114) was effective in reducing MADRS total score after four weeks, with an LS mean change from baseline of 19.8 (SE = 1.3), relative to placebo (*n* = 109) at 15.8 (SE = 1.3). Moreover, in two Phase 3 RCTs, adults with moderate-to-severe MDD (MADRS total score > 28), as well as active suicidal ideation and intent, four weeks of twice-weekly 84 mg intranasal esketamine was superior to placebo in reducing MADRS total score at 24 h post-first dose in patients who received standard of care treatment (initial inpatient psychiatric hospitalization and optimized oral antidepressant). In study 1, esketamine produced an LS mean change from baseline to 24 h post-first dose of 15.9 (*n* = 111, SE = 1.04), relative to placebo nasal spray (*n* = 112, 12.0, SE = 1.02). In study 2, esketamine produced an LS mean change of 16.0 (*n* = 113, SE = 1.02), relative to placebo nasal spray (*n* = 113, 12.2, SE = 1.05) [43, 44].

*Practical Issues and Implementation*: Spravato treatment is provided only in a REMS-certified center, as it requires staff and equipment for patient monitoring after administration.

*Adverse Effects*: The most common adverse reactions (incidence ≥ 5% and at least twice that of placebo plus oral antidepressant) are dissociation, vertigo, sedation, increased blood pressure, anxiety, nausea, hypoesthesia, and vomiting.

#### Primary agents for postpartum depression:

1. Brexanolone (Zulresso)

*Overview*: Brexanolone (Zulresso) is an intravenously administered allosteric modulator of both synaptic and extrasynaptic GABA-A receptors [45]. It was approved in 2019 to treat post-partum depression and might be superior to conventional agents.

*Indications*: Post-partum depression.

*Dose and Route*: Intravenous infusion (continuous) 60–90 µg per kilogram per hour for 60 h.

*Evidence*: The efficacy of brexanolone (Zulresso) in the treatment of postpartum depression (PPD) was evaluated in two Phase 3 randomized controlled trials [46]. In Study 1, patients receiving brexanolone at a target dosage of 90 mcg/kg/hour (*n* = 41) showed a least squares (LS) mean change from baseline in the Hamilton Depression Rating Scale (HAM-D) total score of −17.7 (SE 1.2) at Hour 60, with a placebo-subtracted difference of −3.7 (95% CI: −6.9, −0.5; *p* = 0.0252). At a target dosage of 60 mcg/kg/hour (*n* = 38), the LS mean change was −19.5 (SE 1.2), with a placebo-subtracted difference of −5.5 (95% CI: −8.8, −2.2; *p* = 0.0013). In Study 2, patients receiving brexanolone at a target dosage of 90 mcg/kg/hour (*n* = 51) had an LS mean change from baseline of −14.6 (SE 0.8), with a placebo-subtracted difference of −2.5 (95% CI: −4.5, −0.5; *p* = 0.0160).

*Practical Issues and Implementation*: Brexanolone is a Schedule IV drug available under the Risk Evaluation and Mitigation Strategy (REMS) program. It requires a lengthy infusion time of over 60 h (2.5 days).

*Adverse Effects*: Sedation, dry mouth, loss of consciousness, and flushing.

2. Zuranolone (Zurzuvae)

*Overview*: Zuranolone is an orally administered drug that acts on the GABA-A receptor as a positive allosteric modulator [47–49].

*Indications*: Postpartum depression.

*Dose and Route*: Tablet form. 30 mg to 50 mg once daily dose.

*Evidence*: In a Phase 3 placebo-controlled RCT to assess its efficacy and safety for the treatment of postpartum depression, zuranolone 30 mg demonstrated a significant improvement in depressive symptoms at day 15, with a mean change from baseline in the Hamilton Rating Scale for Depression (HAMD-17) score of −17.8 compared to −13.6 in the placebo group (difference, −4.2; 95% CI, −6.9 to −1.5; *P* = 0.003). Sustained improvements were observed through day 45 [49]. Likewise, in a second Phase 3 RCT, subjects in the zuranolone group (50 mg oral zuranolone once daily for 14 days) experienced statistically significant and sustained improvements in depressive symptoms, with a least squares mean change from baseline in HAMD-17 score of −15.6 at day 15 compared to −11.6 in the placebo group (difference, −4.0; 95% CI, −6.3 to −1.7; *P* < 0.001). Improvements were noted as early as day 3 and were sustained through day 45 [50].

*Practical issues and Implementation*: Can impact a person's ability to drive and perform other potentially hazardous activities. Might cause suicidal thoughts and behavior. Might cause fetal harm.

*Adverse effects*: Drowsiness, dizziness, diarrhea, fatigue, urinary tract infections.

### Detailed descriptions of pipeline medications for depressive disorders (Summarized in Table 4).

**Table 4:** Summary descriptions of the medications in the pipeline for depressive disorders

Name	Mechanism of action	Route and dose	Notes to clinicians (including effects on sedation, weight/lipids, sexual dysfunction and QTc)
Primary Agents for Major Depression			
1. Ammoxetine ([S]−071031B)	Serotonin norepinephrine reuptake inhibitor	Oral, dosing information unknown	Ne reports on sedation, weight/lipid changes, sexual dysfunction, or QTc interval.
2. Navacaprant (BTRX-335140; NMRA-140)	Kappa opioid receptor antagonist	Oral, 80 mg daily	In Phase II trials for mild-to-moderate MDD, improvement was significant at 4 weeks but not at 8 weeks compared to placebo [57].
3. Solriamfetol (Sunosi, SKL-N05)	Dopamine and norepinephrine reuptake inhibitor and trace amine-associated receptor 1 (TAAR1) agonist	Oral, 300 mg daily	FDA approved for excessive daytime sleepiness related to narcolepsy or obstructive sleep apnea. efficacy in (MDD) are pending. Side effects include headache, nausea, decreased appetite, nasopharyngitis, dry mouth, and anxiety.
Augmenting Agents for Major Depression			
1. Aticaprant (JNJ-67953964)	Selective kappa opioid receptor antagonist	Oral, 10 mg daily	No reports on sedation, sexual dysfunction, weight gain or QTc interval activity.
2. Buprenorphine (Subutex)	Partial mu opioid receptor agonist and kappa opioid receptor antagonist	Sublingual 0.4-0.8 mg daily	Studies investigating buprenorphine for treatment of opioid dependence reported sexual dysfunction and premature ejaculation as adverse effects in more than 80% of male participants [106].
3. Celecoxib (CeleBREX; Elyxyb)	Inhibition of cyclooxygenase-2 (COX-2)	Oral, 200-400 mg daily	Risks of cardiovascular events and gastrointestinal bleeding, potential renal and hepatic toxicity. Avoid or use with caution in patients with pre-existing conditions. Consider administering with proton pump inhibitor in patients with GI bleeding risk. Inhibits CYP2D6 and is metabolized by CYP2C9, interacting with enzyme inhibitors.
4. Deuterated Psilocybin Analog (CYB003)	5 HT1A/5 HT2A receptor agonist	Oral, 16 mg (in two sessions)	No results available from clinical trials for CYB003 in depression, though proposed to have a more predictable pharmacokinetic profile compared to psilocybin, with a shorter duration of action.
5. Esmethadone	NMDA receptor antagonist	Oral, 25-50 mg daily	Methadone has previously been observed to contribute to weight gain [107]. Further evaluation of esmethadone on weight gain should be observed.
6. Lumateperone tosylate (Caplyta)	5-HT2A antagonist	Oral, 42 mg daily	Low frequency of sedation, EPS, weight gain, prolactin changes, and QTc prolongation.
7. Milsaperidone (VHX-896)	Metabolite of iloperidone (dopamine D2 and 5-HT2A receptor antagonist)	Oral, dosing information unknown	Possible somnolence, weight gain, and QTc prolongation.
8. Osavampator (NBI-1065845, TAK-653)	AMPA receptor positive allosteric modulator	Oral, dosing information unknown	Somnolence is reported. No reports identified on weight changes, QTc, or sexual dysfunction.
9. Pramipexole	Full dopamine agonist with higher affinity for D3 receptor than D1, D2, and D4 receptors.	Oral, 0.125 mg twice a day	Nausea, dizziness, headache, fatigue, insomnia, constipation, hallucinations, orthostatic hypotension, impulse control disorders (may be less common than in patients with Parkinson disease), and daytime sleepiness.
10. Roflumilast (Daliresp)	Phosphodiesterase-4 inhibitor	Oral, dosing information in depression is unknown.	No reports were identified suggesting sedation, QTc prolongation, or sexual dysfunction. Weight loss is possible.
11. Seltorexant (MIN-202; JNJ-42847922)	Selective orexin-2 receptor antagonist	Oral, 10-40 mg daily	Reports of elevated alanine and aspartate aminotransferase (i.e. affected liver function) as well as insomnia and sleep paralysis have led to discontinuation of participant involvement in some studies [94].
12. Ulotaront (SEP-363856)	Trace-amine associated receptor 1 (TAAR-1) agonist with additional agonism at 5-HT1A	Oral, 25-75 mg daily	No extrapyramidal side effects, weight changes, metabolic effects, alterations in prolactin levels, or changes in QT interval were observed.
Primary Agents for Treatment-Resistant Depression			
1. Psilocybin (COMP360)	5 HT1A/5 HT2A receptor agonist	Oral, 20-30 mg	Studies have shown that psilocybin was effective in decreasing weight gain and obesity trajectory in animal models [108].
Augmenting Agents for Treatment-Resistant Depression			
1. Ketamine (Ketalar)	NMDA receptor antagonist	IV infusion, 0.5 mg/kg over 40 minutes	Use of racemic ketamine has been shown to affect blood pressure and induce hypertension.
2. Minocycline (Minocin)	Tetracycline antibiotic, inhibits bacterial protein synthesis by binding with the 30S and possibly the 50S ribosomal subunit(s), a with pleiotropic anti-neuroinflammatory properties.	Oral, 200 mg daily	Gastrointestinal effects (nausea, vomiting, diarrhea), photosensitivity, skin reactions (pruritus, urticaria, dizziness, vertigo, pseudotumor cerebri autoimmune reactions (drug-induced lupus-like syndrome, autoimmune hepatitis), teeth and bone discoloration, liver toxicity and renal toxicity. Good penetration through the blood-brain barrier, which accounts for its neuroprotective ability.

#### Primary agents for major depression:

1. Ammoxetine ([S]−071031B)

*Overview*: Ammoxetine is a novel SNRI that is currently under investigation for neuropathic pain and depression [51]. It entered a randomized, double-blind, double-dummy, placebo-controlled Phase 3 trial for the treatment of MDD in 2025, with sertraline as an active comparator [52].

*Dose and Route*: Dosing information is unknown.

*Preliminary Findings*: No trials with results investigating the effect of ammoxetine on depression in humans were identified. However, in a rat model of diabetic neuropathic pain, four weeks of ammoxetine treatment reduced depressive behavior and mechanical allodynia [53].

*Adverse Effects*: Nausea, tachycardia, palpitation, xerostomia, dizziness, and pyuria [54].

2. Navacaprant (BTRX-335140; NMRA-140)

*Overview*: Navacaprant is an orally administered selective kappa-opioid receptor antagonist currently being investigated in randomized, double-blind, placebo-controlled Phase 3 trials for MDD [55].

*Dose and Route*: 80 mg once daily oral dose.

*Preliminary Findings*: In a Phase 2 placebo-controlled RCT involving a cohort of patients diagnosed with moderate-to-severe MDD (*n* = 100), the efficacy of once-daily navacaprant monotherapy in alleviating symptoms of depression and anhedonia was investigated, as assessed by the Hamilton Rating Scale for Depression (HAMD-17) and the Snaith-Hamilton Pleasure Scale (SHAPS), respectively. Significant therapeutic benefits were observed with navacaprant treatment compared to placebo. After 4 weeks of treatment, navacaprant produced a reduction of 3.0 points in the Least Squares Mean Difference (LSMD) of the HAMD-17 total score from baseline between navacaprant and placebo groups (*p* = 0.015) and a corresponding reduction of 2.4 points in the SHAPS LSMD (*p* = 0.037). Moreover, after 8 weeks of treatment, the navacaprant group maintained a HAMD-17 LSMD decrease of 2.8 points (*p* = 0.037), as well as a reduction of 4.8 points in the SHAPS LSMD compared to baseline (*p* = 0.001) [56].

*Adverse Effects*: The most common adverse effects reported were headache (5%) and nausea (5%), and those receiving navacaprant reported fewer adverse effects than those receiving placebo (35.3% vs. 44.1%) [57].

1. Solriamfetol (Sunosi, SKL-N05)

*Overview*: Solriamfetol is an orally administered dopamine and norepinephrine reuptake inhibitor and trace amine-associated receptor 1 (TAAR1) agonist, currently approved by the FDA to improve wakefulness in adult patients with excessive daytime sleepiness related to narcolepsy or obstructive sleep apnea [58]. It entered a randomized, double-blind, placebo-controlled Phase 3 trial for the treatment of MDD in 2024 [59].

*Dose and Route*: Oral 300 mg daily.

*Preliminary Findings*: No prior trials were identified investigating the efficacy of solriamfetol in depression.

*Adverse Effects*: Headache, nausea, decreased appetite, nasopharyngitis, dry mouth, and anxiety were reported in the trial for excessive sleepiness in narcolepsy [58].

#### Adjunctive agents for major depression:

1. Aticaprant

*Overview*: Aticaprant is a kappa opioid receptor antagonist that is currently undergoing investigation as an adjunctive therapy for adults with MDD who experience moderate-severe anhedonia and have not responded to other antidepressant treatments [60]. It entered Phase 3 randomized, double-blind, multicenter, parallel-group, placebo-controlled trials for this indication in 2022.

*Dose and Route*: 10 mg daily orally.

*Preliminary Findings*: The efficacy of aticaprant as an adjunctive treatment in major depressive disorder has been evaluated in a large, randomized, double-blind, placebo-controlled phase 2 trial. In this study, 184 adults with MDD and inadequate response to SSRIs or SNRIs were randomized to receive either aticaprant 10 mg daily or placebo for 6 weeks. The primary efficacy endpoint was the change in Montgomery-Åsberg Depression Rating Scale (MADRS) total score. At week 6, the least squares mean difference in MADRS total score for aticaprant versus placebo was significant in both the full intent-to-treat (fITT) population (−3.1, 1-sided *p* = 0.002, effect size 0.36) and the enriched intent-to-treat (eITT) population (−2.1, 1-sided *p* = 0.044, effect size 0.23) [61] (58).

*Adverse Effects*: Common adverse effects include headache, diarrhea, nasopharyngitis, and pruritus [61].

2. Buprenorphine (Subutex)

*Overview*: Buprenorphine is a mu-opioid receptor (MOR) partial agonist and kappa-opioid receptor (KOR) antagonist currently FDA-approved for the treatment of opioid dependence. It is currently undergoing Phase 3 randomized, placebo-controlled, parallel-group trials as an adjunctive treatment to reduce the occurrence of severe suicidal ideation in major depression [62].

*Dose and Route*: 0.4 or 0.8 mg daily sublingually.

*Preliminary Findings*: In a phase 2 RCT of severely suicidal patients without a history of substance abuse, ultra-low-dose adjunctive buprenorphine was associated with decreased suicidal ideation relative to placebo, based on the Beck Suicide Ideation Scale after both two (mean difference −4.3, 95% CL = −8.5, −0.2) and four weeks (mean difference −7.1, 95% CL = −12.0, −2.3) of treatment. No withdrawal symptoms were reported after discontinuation of buprenorphine [63].

*Adverse Effects*: Common adverse effects include anticholinergic effects, including some extent of QT prolongation, as well as hypotension, headache, constipation, and nausea.

3. Celecoxib (Celebrex)

*Overview*: Celecoxib is a COX-2 inhibitor and nonsteroidal anti-inflammatory drug (NSAID). Contains a sulfonamide moiety and may cause allergic reactions in those allergic to other sulfonamide-containing drugs. It is currently undergoing Phase 3, randomized, double-blind, placebo-controlled trials for the treatment of MDD [64].

*Dose and Route*: Oral tablet. Celecoxib in a dose of 200 to 400 mg/day was used for 6 weeks.

*Preliminary findings*: Abbasi et al. evaluated the efficacy of celecoxib as an adjunctive treatment in patients with major depressive disorder (MDD). In this study, 40 patients with MDD were randomized to receive either celecoxib (200 mg twice daily) or placebo in addition to sertraline (200 mg/day) for 6 weeks. The primary outcomes were changes in Hamilton Depression Rating Scale (Ham-D) scores and serum IL-6 concentrations. The results demonstrated that the celecoxib group had a significantly greater reduction in Ham-D scores compared to the placebo group (mean difference = 3.35, 95% CI: 1.08 to 5.61, *P* = 0.005). Additionally, the celecoxib group showed a higher response rate (95% vs. 50%, *P* = 0.003) and remission rate (35% vs. 5%, *P* = 0.04) compared to the placebo group. The reduction in depressive symptoms was significantly correlated with the reduction in serum IL-6 levels (*r* = 0.673, *P* < 0.001) [65].

*Adverse effects*: Cardiovascular thrombotic events like MI and stroke, GI side effects like gastric mucous ulceration, and anemia.

4. Deuterated Psilocybin Analog (CYB003)

*Overview*: As an orally administered, deuterated analog of psilocybin, CYB003 is a psychedelic and potent biased agonist of the serotonin 5-HT2A receptor. It entered Phase 3 randomized, double-blind, placebo-controlled clinical trials for the adjunctive treatment of MDD in November 2024 [66].

*Dose and Route*: 16 mg by mouth in two dosing sessions.

*Preliminary Findings*: A Phase 1/2 randomized, double-blind, placebo-controlled trial was completed in January 2024 to determine the safety and tolerability of ascending oral doses of CYB003 in healthy participants with and without MDD. Results are currently pending [67].

*Adverse Effects*: No data was found regarding the adverse effects of the deuterated analog of psilocybin specifically; however, it may be reasonable to infer that its adverse effect profile aligns with that of psilocybin. Commonly reported adverse effects associated with psilocybin include headache, as well as physical and emotional discomfort [68].

5. Esmethadone (REL1017, XEN1101)

*Overview*: Esmethadone is an orally administered NMDA receptor antagonist currently undergoing a Phase 3 trial for use in major depression [69, 70]. It has completed a randomized, double-blind, placebo-controlled Phase 3 after demonstrating positive results in a Phase 2 trial.

*Dose and Route*: Esmethadone oral tablet 25 mg to 50 mg daily.

*Preliminary Findings*: The efficacy of esmethadone (REL-1017) as an adjunctive treatment in major depressive disorder (MDD) has been evaluated in a Phase 3 RCT. In this trial, outpatients with MDD and inadequate response to standard antidepressants were randomized to receive either esmethadone or placebo. The primary efficacy measure was the change from baseline to day 28 in the MADRS score. The intent-to-treat (ITT) analysis showed a mean change from baseline (CFB) of 15.1 (SD 11.3) for esmethadone and 12.9 (SD 10.4) for placebo, with a mean difference (MD) of 2.3, which was not statistically significant (*P* = 0.154; Cohen effect size [ES] = 0.21). Remission rates were 22.1% for esmethadone and 13.2% for placebo (*P* = 0.076), and response rates were 39.8% and 27.2% (P = 0.044), respectively. In the per-protocol (PP) analysis, the mean CFB was 15.6 (SD 11.2) for esmethadone and 12.5 (SD 9.9) for placebo, with an MD of 3.1 (*P* = 0.051; ES = 0.29) [70]. Nevertheless, Phase 3 trials investigating adjunctive esmethadone in MDD are ongoing, with an estimated completion in December 2026 [71].

*Adverse Effects*: headache, constipation, nausea, somnolence, dizziness.

1. Lumateperone tosylate (Caplyta)

*Overview*: Lumateperone is an atypical antipsychotic that is a 5-HT2A receptor antagonist. It is FDA-approved for schizophrenia and bipolar depression (as monotherapy and adjunctive therapy). It has entered Phase 3 randomized, double-blind, placebo-controlled clinical trials as an adjunct to conventional antidepressants for MDD [72].

*Dose and Route*: Initial dose 42 mg once daily.

*Preliminary Findings*: Several Phase 3 randomized, double-blind, placebo-controlled trials were completed in 2024 to determine the efficacy of lumateperone as adjunctive therapy in the treatment of MDD; however, the results of these studies were not identified [72–74]. At the time of publication, an additional Phase 3 trial of lumateperone adjunctive therapy for MDD is underway, with an estimated completion by the end of 2026 [75].

*Adverse Effects*: Commonly reported adverse effects include somnolence, nausea, and dizziness.

7. Milsaperidone (VHX-896)

*Overview*: Milsaperidone is an active metabolite of iloperidone (Fanapt), an atypical antipsychotic approved for the treatment of schizophrenia and bipolar I disorder in adults [76–78]. It entered randomized, double-blind, placebo-controlled multicenter Phase 3 clinical trials for the adjunctive treatment of MDD in early 2025 [79].

*Dose and Route*: Oral route. Dosing information is unknown.

*Preliminary Findings*: No trials were identified investigating the effect of milsaperidone on unipolar depression in humans.

*Adverse Effects*: No identified trials reported on the adverse effects of milsaperidone in particular; however, iloperidone is known to produce tachycardia, dizziness, xerostomia, nasal congestion, weight gain, and somnolence [80].

8. Osavampator (NBI-1065845, TAK-653)

*Overview:* Osavampator is a positive allosteric modulator at the AMPA receptor, which entered Phase 3 randomized, double-blind, placebo-controlled clinical trials for the adjunctive treatment of MDD in early 2025 [81–83].

*Dose and Route:* Oral route. Dosing information is unknown.

*Preliminary findings*: No trials were identified investigating the effect of osavampator on depression in humans. However, 6 days of osavampator treatment produced antidepressant-like effects in the reduction of submissive behavior model in rats without inducing hyperlocomotor behavior [84].

*Adverse effects*: Somnolence, headache, and nasopharyngitis [85].

9. Pramipexole

*Overview*: Pramipexole is a dopamine receptor agonist approved for the treatment of Parkinson’s disease and restless legs syndrome. Its high affinity for the D3 receptor and neuroprotective, antioxidant, and anti-inflammatory activity provides a rationale for the treatment of depression. Phase 2 clinical trials have shown promising results in the treatment of MDD, and Phase 3 randomized double-blind, as well as open-label follow-up trials, are in progress [86–88].

*Dose and Route*: Oral tablet. 0.125 mg twice a day.

*Preliminary findings*: In an 8-week, double-blind, placebo-controlled study, 60 outpatients with nonpsychotic MDD were randomized to receive either pramipexole (*n* = 30) or placebo (*n* = 30) in addition to their standard antidepressant treatment. Utilizing MADRS score as the primary outcome measure, the mixed-effects linear regression model analysis showed a modest but statistically significant benefit of pramipexole (*P* = 0.038). The last-observation-carried-forward analyses indicated that 40% of patients in the pramipexole group achieved response compared to 27% in the placebo group (χ^2^ = 1.2, *P* = 0.27), and 33% achieved remission compared to 23% in the placebo group (χ^2^ = 0.74, *P* = 0.61), though these differences were not statistically significant [87].

*Adverse effects*: Headache, peripheral edema, hyperalgesia, impulsive compulsive behaviors, orthostatic hypotension, hallucinations, twitching.

10. Roflumilast (Daliresp)

*Overview*: Roflumilast is a selective phosphodiesterase-4 (PDE4) inhibitor approved for the treatment of chronic obstructive pulmonary disease (COPD) [89]. It entered Phase 3 randomized, placebo-controlled, double-blind, parallel-group trials for the adjunctive treatment of MDD in early 2025 [90].

*Dose and Route*: Oral tablet. Dosing information is unknown.

*Preliminary findings*: No trials were identified investigating the effect of roflumilast on depression in humans. However, in an experimental autoimmune encephalomyelitis rat model, roflumilast improved symptoms of depression and motor dysfunction, in addition to reducing neuroinflammation and suppressing microglial activation [89].

*Adverse effects*: Gastrointestinal symptoms, loss of appetite, weight loss, itching, and headache [91].

11. Seltorexant (MIN-202; JNJ-42847922)

*Overview*: Seltorexant is an orally administered selective orexin-2 receptor antagonist. It is undergoing development as an adjunctive therapy with antidepressants for adult and elderly patients with MDD and insomnia [92]. Phase 3 study results from randomized, multicenter, double-blind, placebo-controlled trials are pending [93].

*Dose and Route*: Oral tablet 10 mg to 40 mg daily.

*Preliminary Findings*: In a Phase 2b, placebo-controlled RCT, seltorexant was evaluated as an adjunctive therapy for the treatment of MDD. Patients were randomized to receive either a placebo or seltorexant (20 mg or 40 mg) once daily, adjunctive to their current antidepressant. The primary endpoint was the change from baseline in the MADRS total score at week 6. The results demonstrated a significant improvement in MADRS total score for the seltorexant 20 mg group compared to placebo at week 3 (least-square means difference: −4.5, 90% CI: −6.96 to −2.07, *P* = 0.003) and a trend towards significance at week 6 (least-square means difference: −3.1, 90% CI: −6.13 to −0.16, *P* = 0.083). Notably, patients with higher baseline insomnia severity (Insomnia Severity Index ≥ 15) showed a greater treatment effect with seltorexant 20 mg compared to placebo (least-square means difference: −4.9, 90% CI: −8.98 to −0.80) [94].

*Adverse Effects*: Somnolence, headache, nausea.

12. Ulotaront (SEP-363856)

*Overview*: Ulotaront is an orally administered trace amine-associated receptor 1 and serotonin 5-HT1A receptor agonist. As an investigational antipsychotic, in 2023, it failed to meet its primary endpoint in Phase 3 trials for schizophrenia after being granted an FDA Breakthrough Therapy Designation. At present, it is in Phase 2/3, multicenter, randomized, double-blind, placebo-controlled clinical trials for the adjunctive treatment of major depression in adults [95, 96].

*Dose and Route*: Oral tablet or capsule form. 25 to 75 mg daily dose.

*Preliminary findings*: No prior trials were identified investigating the efficacy of ulotaront in the treatment of depression.

*Adverse effects*: Somnolence, nausea, dizziness, dry mouth, headache.

#### Primary agents for treatment-resistant depression:

1. Psilocybin (COMP360)

*Overview*: Psilocybin is an orally administered, serotonin 5-HT2A and 5-HT1A agonist that was granted FDA Breakthrough Therapy designation for depression in 2018 and a second time in 2019 [68, 97]. Psilocybin has recently been studied for treatment-resistant depression, anxiety related to end-stage illness, and substance use disorders with favorable results. It is currently classified as a Schedule I substance under the Controlled Substances Act. Psilocybin is currently in Phase 3 randomized, multicenter, double-blind, placebo-controlled trials investigating the safety and efficacy of both one-time and repeat doses for treatment-resistant depression [97].

*Dose and Route*: Oral 20 to 30 mg.

*Preliminary Findings*: In a Phase 2 double-blind RCT, the efficacy of a single dose of psilocybin (25 mg) was evaluated in patients with treatment-resistant major depression. The study included 233 participants who were randomized to receive either 25 mg (*n* = 79), 10 mg (*n* = 75), or 1 mg (control, *n* = 79) of psilocybin. The primary outcome was the change in the MADRS score from baseline to week 3. The results showed a significant reduction in MADRS scores in the 25 mg group compared to the 1 mg group, with a least-squares mean change of −12.0 versus −5.4, respectively (difference of −6.6; 95% CI, −10.2 to −2.9; *P* < 0.001). Unlike the 25 mg dose, the 10 mg dose was not found superior to the control group, with a least-squares mean change from baseline of −7.9 (difference of −2.5; 95% Cl, −6.2 to 1.2; *P* = 0.18) [98].

*Adverse Events*: Commonly reported adverse events included headache, as well as physical and emotional discomfort.

#### Augmenting agents for treatment-resistant depression:

1. Ketamine (Ketalar)

*Overview*: Ketamine is an NMDA receptor antagonist and dissociative anesthetic used for the induction and maintenance of anesthesia. The (S) enantiomer of ketamine, known as esketamine, was approved by the FDA for TRD, as well as MDD accompanied by suicidal thoughts or actions, in 2019. Racemic ketamine, typically delivered through an IV, is undergoing a number of Phase 3 clinical trials for the treatment of TRD and suicidality in MDD [99–102].

*Dose and Route*: IV infusion. In clinical trials, it has consisted of 0.5 mg/kg over 40 min.

*Preliminary findings*: Murrough et al. evaluated the efficacy of intravenous ketamine in treatment-resistant major depression. In a two-site, parallel-arm study, 73 patients were randomized to receive a single infusion of ketamine (0.5 mg/kg) or midazolam (active placebo) in a 2:1 ratio. The primary outcome was the change in MADRS scores 24 h post-infusion. The ketamine group had a significantly greater reduction in MADRS scores compared to the midazolam group, with a mean difference of 7.95 points (95% CI, 3.20 to 12.71). The response rate at 24 h was 64% for ketamine versus 28% for midazolam, with an odds ratio of 2.18 (95% CI, 1.21 to 4.14) [103].

*Adverse effects*: Common adverse effects include dissociation, vertigo, sedation, hypertension, anxiety, nausea, hypoesthesia, and vomiting.

2. Minocycline (Minocin)

*Overview*: Minocycline is an antibiotic with anti-inflammatory, antioxidant, and neuroprotective properties. Minocycline might improve depressive symptoms and augment response to treatment in patients with depression, irrespective of treatment resistance. It is currently undergoing Phase 3 randomized, double-blind, placebo-controlled trials as an adjunctive treatment for treatment-resistant depression [104].

*Dose and Route*: Oral tablet. 200 mg daily.

*Preliminary findings*: In a phase 2 placebo-controlled RCT investigating the efficacy of 6 weeks of adjunctive minocycline in TRD, minocycline was not superior to placebo in attenuating symptoms of depression, as indicated by a statistically significant change in MADRS scores from baseline to endpoint compared to placebo [105]. Nevertheless, phase 3 trials are in progress with an estimated completion in early 2025.

*Adverse effects*: Diarrhea, dizziness, unsteadiness, drowsiness, mouth sores, headache, vomiting, increased sensitivity to sunlight, affects the quality of sleep, and lupus.

## Discussion

This systematic review has identified the FDA-approved medications for the treatment of depressive disorders, as well as those in the pipeline between 2009 and April 1, 2025. Over this period, there has been significant progress in the development of pharmacotherapies for depression, reflecting ongoing efforts to address the unmet needs of patients. The medications have been listed alphabetically by indication, alongside evidence for their use, practical and implementational considerations, and their adverse effects. During this period, 15 medications have been approved by the FDA for the treatment of depressive disorders, and 18 have entered Phase 3 clinical trials to investigate their effects as potential antidepressants.

Notably, during this period, we find a remarkable number of approved and Phase 3 pipeline antidepressants whose mechanisms deviate from the monoamine hypothesis, interacting with several other, non-monoamine neurotransmitter systems. In particular, we witnessed the introduction of antidepressants, which act as antagonists at the glutamatergic NMDA receptor, which in recent years has been the target of much research [109].

In 2020, esketamine nasal spray (Spravato) was approved by the FDA as an adjunctive therapy for treatment-resistant depression in adults, as well as MDD (MDD) accompanied by depressive symptoms and acute suicidal ideation or behavior. While esketamine remains underutilized by clinicians at this time, possibly due to its cost and restrictions imposed by the REMS program [110], it is particularly significant, as its rapid onset of action in attenuating symptoms of depression (of 4 h), as well as efficacy in, render it an ideal choice for hospitalized patients and patients waiting for another oral antidepressant to take effect [44]. Long-term outcomes of continued esketamine treatment for TRD appear superior to placebo as adjunctive to traditional antidepressants, though further research is needed [111]. In a phase 3, multicenter, double-blind, randomized trial (*n* = 297), continued adjunctive treatment with esketamine after 16 weeks of initial treatment was significantly superior to placebo in relapse prevention among patients with TRD [111]. Spravato is undergoing FDA review for approval as monotherapy for treatment-resistant depression in Phase 4 trials [112], and a recent randomized clinical trial showed its non-inferiority to ECT in TRD [113].

The 2022 U.S. Department of Veterans Affairs and U.S. Department of Defense (VA/DoD) Clinical Practice Guideline for the Management of Major Depressive Disorder includes several significant updates. In particular, it now suggests esketamine or ketamine as treatment options for patients who have not responded to several adequate pharmacologic trials [114]. This is a notable change from the 2016 guideline, which recommended against the use of ketamine outside research settings due to a gap in knowledge. Esketamine was not included in the 2016 guideline as it was not commercially available at that time [114].

In 2023, a combination formulation of dextromethorphan plus bupropion (Auvelity) was approved, after receiving a Breakthrough Therapy designation by the FDA in 2019. As an uncompetitive NMDA receptor antagonist, dextromethorphan (DXM) is coupled with bupropion, which functions as a CYP2D6 inhibitor (to prolong the relatively rapid metabolism of DXM) and norepinephrine-dopamine reuptake inhibitor [41]. As the first oral antidepressant with a new mechanism of action approved by the FDA for MDD in over 60 years, the efficacy of dextromethorphan in MDD represents a significant deviation from the classic monoaminergic hypothesis that is representative of our evolving understanding of the pathophysiology of depression.

Esmethadone, the S-enantiomer of methadone, is currently in Phase 3 clinical trials, both as an adjunct and as monotherapy for the treatment of MDD. Despite its structural/enantiomeric similarity to racemic methadone, it acts primarily at NMDA receptors and exhibits a relative lack of activity at opioid receptors, relative to (R)-. This has been confirmed by in vitro and animal, and human studies showing no meaningful abuse potential [69]. Moreover, clinical trials are underway for intravenous racemic ketamine, which is four times less potent than esketamine and is possibly more effective than intranasal esketamine in attenuating symptoms of depression [115]. In a recent retrospective study in patients with treatment-resistant bipolar depression, IV ketamine was well-tolerated and produced significant reductions on the MADRS without precipitating manic switches in any patients during the treatment period [116]. Given that ketamine and other NMDA receptor antagonists produce an activity-dependent release of brain-derived neurotrophic factor (BDNF), which acts as a pivotal mediator of synaptic plasticity [117], our findings illustrate a shift from the traditional monoamine hypothesis to a neuroplasticity hypothesis of depression, reflected in the landscape of pharmacotherapy for depression over the past fifteen years.

NMDA receptor antagonists may offer alternative treatment options for patients who do not respond adequately to conventional antidepressants, thereby expanding the therapeutic armamentarium available to clinicians. By targeting the glutamatergic system and helping to mediate synaptic plasticity, these drugs seem to address the limitations of existing treatments and provide new avenues for managing depressive symptoms. Despite this, the real-world accessibility of NMDA receptor modulators remains a challenge. While their use is expanding as they become integrated into clinical practice guidelines, significant barriers exist to their implementation, including cost, travel distance to specialized infusion centers, REMS programs mandated by the FDA, and insurance coverage, among others [118]. Alternative care models may help to mitigate these challenges and improve patient access to these emerging treatments [118, 119].

In addition to NMDA antagonism, between 2009 and early 2025, several novel drugs that act as allosteric modulators at the GABA-A receptor were approved by the FDA. Brexanolone (Zulresso) and zuranolone (Zurzuvae), both analogs of allopregnanolone, are newly approved for the treatment of postpartum depression (PPD) in adults. Before the approval of these medications, there were limited treatment options specifically available for this indication, leaving many women without adequate relief from their symptoms. PPD is the most common complication of childbirth, affecting 10–15% of women [120], which is an area of significant unmet need. For instance, less than 20% of women receive treatment for PPD, mostly with a slow onset of action and inadequate response. Poorly treated or untreated PPD places women at risk for impaired bonding with the newborn, impeded cognitive and emotional development of the infant, marital discord, and maternal suicide [120]. Therefore, brexanolone and zuranolone represent a significant breakthrough in maternal mental health care. The American College of Obstetricians and Gynecologists (ACOG) recommends brexanolone for moderate-to-severe perinatal depression with onset in the third trimester or within four weeks postpartum, emphasizing its rapid onset of action [121]. However, its use is limited by the need for a 60-h intravenous infusion and inpatient monitoring due to risks such as excessive sedation and loss of consciousness. Zuranolone, approved in 2023, offers a significant advancement as the first oral treatment approved for PPD. This medication is administered as a 14-day course and has shown efficacy in reducing depressive symptoms rapidly, with sustained effects observed up to 45 days post-treatment. The oral administration of zuranolone allows for outpatient treatment, which is a considerable advantage over brexanolone's intravenous route. The ACOG has since published a Practice Advisory to recommend consideration of zuranolone in the postpartum period for depression that has onset in the third trimester or within four weeks postpartum [122].

Several drugs that act as antagonists at the kappa-opioid receptor (KOR) have undergone Phase 3 trials to assess their efficacy in depressive disorders. Navacaprant and aticaprant are both highly selective KOR antagonists currently in Phase 3 clinical trials to assess efficacy in MDD. Aticaprant, a highly selective KOR antagonist, is currently in Phase 3 trials to assess efficacy in adults with MDD accompanied by moderate-to-severe anhedonia who have not adequately responded to current antidepressant therapy with an SSRI or SNRI. Another marker of this trend is the FORWARD-3 trial of buprenorphine (mu-opioid receptor partial agonist and kappa opioid receptor antagonist) plus samidorphan (mu-opioid receptor antagonist) in MDD, which did not outperform placebo, despite positive phase 2 study results. Given the substantial body of evidence suggesting the involvement of the opioid system in the pathophysiology of depression [123], ongoing research into novel KOR antagonists and compounds targeting the opioid system represents a promising avenue for the development of new antidepressants.

During this period, we additionally saw the first Phase 3 clinical trial of psychedelics in depression. In 2022, psilocybin (COMP360), a psychedelic that acts as a biased agonist at the 5-HT2A receptor, entered Phase 3 clinical trials to assess efficacy, safety, and tolerability in patients with treatment-resistant depression. Phase 2 trials of psilocybin demonstrated promising results, indicating significant reductions in depressive symptoms and improvements in mood and overall well-being following psychedelic-assisted therapy sessions [68]. While at this time, the current state of research is not sufficient to warrant FDA approval of psychedelics for depressive disorders, recent Phase 3 clinical trials of psilocybin, if positive, pave the way for psychedelics to enter clinical practice in the treatment of depression [124, 125].

We also saw the development of other novel antidepressants with primarily monoaminergic-based mechanisms of action, which followed several notable trends. In particular, several newer antidepressants acted as partial agonists at the 5-HT1A receptor. Recent significant emphasis on the 5-HT1A receptor further reflects an evolving understanding of the pathophysiology of depression. For example, it is known that chronic treatment with SSRIs and 5-HT1A receptor agonists produces desensitization of 5-HT1A autoreceptors in the raphe nucleus that permits increased 5-HT neurotransmission, which may underlie the anti-depressive and anxiolytic effects [126]. In 2023, gepirone was approved by the FDA for the treatment of MDD. As a selective partial agonist at the 5-HT1A receptor, gepirone (Exxua) is one of two azapirones available in the US and the only azapirone medication indicated for MDD [12].

Moreover, vilazodone (Viibryd) acts both as a partial agonist at the 5-HT1A receptor and as an inhibitor at the serotonin transporter (SERT). Relative to SSRIs alone, vilazodone’s combined mechanism may decrease time to efficacy, reduce the incidence of sexual side effects, and provide concomitant anxiolytic properties [127, 128]. Lastly, vortioxetine (Brintellix), which acts as a SERT inhibitor, agonist at the 5-HT1A receptor, partial agonist at the 5-HT1B receptor, and antagonist at the 5-HT3, 5-HT1D, and 5-HT7 receptors, was approved by the FDA for the treatment of MDD in 2013. Both vilazodone and vortioxetine are recommended by the 2022 VA/DoD Clinical Practice Guideline for the Management of Major Depressive Disorder as initial pharmacotherapy, alongside SSRIs, SNRIs, bupropion, mirtazapine, and trazodone [114].

Solriamfetol (Sunosi), currently available for the treatment of excessive daytime sleepiness associated with narcolepsy and obstructive sleep apnea, entered phase 3 clinical trials for depression in 2024, acting as a dopamine and norepinephrine reuptake inhibitor and trace amine-associated receptor 1 (TAAR1) agonist. TAAR1 is a Gs-coupled receptor that may heterodimerize with D2 receptors, and when agonized, typically exerts inhibitory effects on dopaminergic neurotransmission [129]. With its combined dopamine and norepinephrine reuptake inhibition, as well as TAAR1 agonism, solriamfetol provides a novel mechanism of action for the treatment of depression, possibly offering an effective alternative for patients who do not respond well to traditional antidepressants.

Additionally, during this period, a relatively large number of atypical antipsychotics were approved and were in Phase 3 clinical trials for depressive disorders. From 2009 through early 2025, three antipsychotics were approved for the adjunctive treatment of MDD, including brexpiprazole (Rexulti), cariprazine (Vraylar), and quetiapine extended-release (Seroquel XR). Such drugs may be particularly significant for patients exhibiting treatment resistance and/or mixed features, providing clinicians with more tools to tailor pharmacotherapy to individual patient needs.

Several novel medications under investigation in patients with depression were at other phases of the pipeline or not registered in the U.S. Clinical Trials Registry during the study inclusion period and thus were excluded from this review. For instance, liraglutide, a glucagon-like peptide-1 (GLP-1) receptor agonist which was found efficacious in individuals with MDD or bipolar disorder and obesity, with a 180-day follow-up period, was not identified in the initial search [130]. Liraglutide is currently FDA-approved for chronic weight management as an adjunct to a reduced-calorie diet and increased physical activity [131]. GLP-1 receptor agonists have been investigated with some efficacy in mood disorders, and further research is needed to ascertain any potential role in treating these debilitating conditions [132].

Notably, Phase 4 post-market surveillance trials, designed to reveal the longer-term safety, tolerability, and efficacy of approved drugs, were not included to maintain a focus on emerging therapeutics closer to market. For example, Phase 4 trials of tianeptine, an atypical tricyclic antidepressant currently approved in parts of Europe, Asia, and Latin America, began in 2020 [133, 134]. Despite its tricyclic structure, tianeptine functions as an agonist at the mu and delta-opioid receptors, with no other known receptor targets [134], and seems at least as efficacious as SSRIs in the treatment of depression, with a greater tolerability profile [135].

Additionally, many unique agents in early-stage clinical trials were excluded from this review. In a Phase 1/2 trial conducted in 2023, GH-001, a vaporized formulation of 5-methoxy-N, N-dimethyltryptamine (5-MeO-DMT/mebufotenin), was well-tolerated and demonstrated rapid antidepressant effects on the MADRS in patients with treatment-resistant depression [136]. Further investigation is ongoing with GH-001 and other psychedelics in the treatment of depressive disorders. Furthermore, an oral formulation of brexanolone designed for two or three times daily administration for two days, by the name of LPCN-1154, is currently undergoing Phase 2 trials for the treatment of postpartum depression after positive results in a pharmacokinetic dose proportionality study [137]. Future research may explore antidepressant therapies at other stages of the pipeline to assess how emerging and established antidepressants might be tailored to individual patient profiles.

We found that every oral antidepressant approved by the FDA from 2009 through early 2025 can be administered once daily, with the exception of Auvelity (dextromethorphan plus bupropion), representing a major shift toward extended and delayed-release preparations. Notably, we see several extended-release formulations of previously approved medications designed to simplify patient medication adherence. A new formulation of once-per-day extended-release bupropion was approved (Forfivo XL). We additionally see the introduction of a delayed-release duloxetine formulation (Drizalma Sprinkle) in 2019 and an extended-release trazodone in 2010 (Oleptro). In addition to streamlining patient adherence to treatment regimens, this shift toward once-daily dosing helps to increase tolerability by minimizing fluctuations in drug concentrations throughout the duration of action, reducing the occurrence of pharmacokinetic peaks and troughs associated with immediate-release formulations [138]. This aligns with broader trends in psychiatry favoring the development of longer-acting medications, exemplified by the relatively recent introduction of long-acting antipsychotics, with the longest-acting formulation (Invega Hafyera) requiring administration only once every six months [139].

The year 2024 also witnessed the introduction of an FDA-approved depression App called Rejoyn as an adjunctive treatment to antidepressants. Rejoyn, which is available by prescription, provides “CBT-based lessons, emotional faces memory task exercises, and personalized reminders and messaging” over six weeks, with an additional 4-week availability of CBT [140]. Rejoyn was approved based on a 13-week double-blind, randomized, controlled trial compared to a sham app, with nearly 400 patients showing efficacy as evidenced by the MADRS [141].

Lastly, prior to 2009, nearly every antidepressant on the market functioned primarily via a monoaminergic mechanism of action, which may be designated first generation antidepressants (e.g., tricyclic antidepressants and MAOIs) and second-generation antidepressants (e.g., SSRIs and SNRIs) [142]. However, as of early 2025, a substantial number of non-monoaminergic, ‘third-generation’ antidepressants emerged, many of which have already received FDA approval or entered Phase 3 clinical trials, a process which often requires a decade or longer of preclinical and clinical research. Given that 30% or more of individuals with depression exhibit resistance to conventional pharmacological therapies [3], the emergence of third-generation antidepressants may substantially improve the lives of many suffering from this debilitating condition.

Treatment resistance in major depression is influenced by biological, psychological, and social factors [143]. Childhood trauma is a well-documented risk factor and predictor of response to antidepressants [144]. In a large RCT investigating optimal predictors of treatment for depression (*n* = 1008), individuals with a history of abuse between the ages of 4 to 7 years were 1.6 times less likely to achieve response or remission with serotonergic antidepressants [144]. Psychiatric and physical comorbidities, including anxiety disorders, substance use disorders, personality disorders, and chronic pain, further reduce treatment outcomes in depression [145–147]. Moreover, social determinants, including financial strain, neighborhood income, and interpersonal conflict, are associated with poorer antidepressant outcomes and higher depression severity [148–151]. Of note, lower socioeconomic status is associated with poorer antidepressant treatment outcomes even in the context of clinical trials in which all participants receive equal access to high-quality, uniform care [152].

Pharmacogenomic testing has been proposed as a tool to guide antidepressant selection [153]. At present, the major focus has been on assessing pharmacokinetic gene variants, particularly the cytochrome P450 family of enzymes (i.e., CYP2D6 and CYP2C19), which influence the metabolism of many antidepressants [154, 155]. Such testing has the potential to reduce the prescription of medications with predicted drug-gene interactions, though in its current stage does not appear to carry consistent effects on symptom remission [156]. As novel third-generation antidepressants emerge from the pipeline, future research is needed to evaluate the effect of pharmacogenomically-guided antidepressant selection on patient outcomes.

Moreover, brain stimulation techniques such as repetitive transcranial magnetic stimulation (rTMS) and electroconvulsive therapy (ECT) have demonstrated efficacy in TRD [157, 158]. ECT is particularly effective, with response rates ranging from 50–70% of patients, relative to rTMS, with response rates around 30–40% [157, 159–161]. Despite the availability of these evidence-based interventions, several barriers hamper their widespread uptake into clinical practice. High costs, limited insurance coverage, geographic disparities in provider availability, limited provider knowledge, and stigma contribute to underutilization [162, 163]. Additionally, frequent treatment sessions are required for both rTMS, which typically necessitates daily administration for 4–6 weeks, and ECT, which often requires maintenance sessions, further limiting their accessibility [164–166].

Long-term outcomes for TRD remain poor, with relapse rates of up to 80% within only a year of achieving remission [167]. The rate of recovery within 10 years is approximately 40%, and TRD is associated with substantial loss in quality of life, as well as significant functional impairment, morbidity, and mortality [167–170]. Individuals with TRD have a nearly twofold higher risk of suicide relative to those with non-TRD major depression [170–172]. Given the high burden of TRD on affected individuals, there is an urgent need for more effective treatment strategies. We identified significant advancements in the development of novel antidepressants with non-monoaminergic mechanisms, including many targeting glutamatergic, neuropeptide, and anti-inflammatory pathways. However, whether these agents reduce the incidence of treatment resistance or exhibit superior efficacy in head-to-head comparisons with traditional monoaminergic antidepressants remains to be definitively established. While promising, further rigorous clinical trials are needed to assess their longer-term efficacy in the management of TRD and establish their role in the clinical répertoire.

This review is subject to several limitations. Firstly, the search criteria relied on publicly available databases, which may not capture all relevant medications, such as those undergoing trials outside the U.S. Additionally, the results of clinical trials with negative or non-significant results are less likely to be published or may be selectively reported, leading to potential publication bias. Furthermore, the exclusion of suspended, withdrawn, or terminated trials, as well as those that did not meet their primary endpoint, may have introduced selection bias by omitting potentially informative studies. Clinical trials reporting negative or non-significant results are also underrepresented due to publication bias, which could skew our findings toward more favorable outcomes. The search strategy was also limited by language restrictions, as the databases used primarily include records published in English. Lastly, the heterogeneity of identified clinical trials, including variations in study populations, outcome measures, and duration, limits the direct comparability of their findings. Further research is needed to evaluate and compare the efficacy of novel drugs for treating depression in the population at large.

## Conclusion

From 2009 through early 2025, remarkable advancements have been made in the development of novel third-generation antidepressants, representing a marked shift away from the monoaminergic hypothesis of depression. Several trends were identified in the mechanisms of FDA-approved drugs and those in Phase 3 trials for depressive disorders during this period, including NMDA receptor antagonism, kappa-opioid receptor antagonism, 5-HT1A receptor partial agonism, serotonin-norepinephrine reuptake inhibition, and the use of atypical antipsychotics as an adjunct to treatment with classical antidepressants. These therapies show promising potential in addressing TRD and provide novel options for patients who have not responded to traditional antidepressants. Moreover, nearly every oral antidepressant we found that was FDA-approved during this time has a once-daily dosing schedule, reflecting a broader effort to improve patient adherence through extended and delayed release formulations. Future research should investigate the comparative long-term efficacy and safety of novel third-generation agents relative to traditional monoaminergic agents, particularly in TRD.

## Supplementary Information

Supplementary Material 1.

Supplementary Material 2.

## Data Availability

No datasets were generated or analysed during the current study.

## Abbreviations

FDA: United States Food and Drug Administration
NMDA: N-methyl-D-aspartate
GABA-A: Gamma-aminobutyric acid type A
5-HT1A: 5-Hydroxytryptamine (serotonin) type 1A
MDD: Major depressive disorder
