# Supplementary material for: Depressive disorders: systematic review of approved psychiatric medications (2009-April 2025) and pipeline phase 3 medications
Source: BMC Psychiatry. 2025 Oct 7;25:939. doi: 10.1186/s12888-025-07141-3 (PMC12506068; doi:10.1186/s12888-025-07141-3)
Supplement: Supplementary file 1 — Supplementary Material 1. [file 12888_2025_7141_MOESM1_ESM.docx]

**Supplementary File: Full Search Strings**

**Databases Searched:**

1. FDA Label Database
2. ClinicalTrials.gov

**Date of Last Searches:** April 2, 2025

1. **FDALabel Database search**

**Parameters:**

- 1. Drug Type: “Human Prescription Drug”
  2. Application: “NDA” (New Drug Application)
  3. Labeling (Indications & Usage): “depression OR major depressive disorder”
  4. Market Status: “Active” between “01/01/2009” and “04/01/2025”.

Records identified from search: 78

1. **ClinicalTrials.gov search**

**Parameters:**

- 1. Phase: “Phase III”
  2. Conditions: “depression OR major depressive disorder”
  3. Intervention: “drug”
  4. Study start: from “01/01/2009” to “04/01/2025”

Records identified from search: 423

**PRISMA flow diagram of included novel antidepressants from clinical trials and FDA approval data**

**Identification of novel compounds from clinical trials and FDA approvals**

Records identified from:

FDALabel Database search with specified criteria (n = 78)

ClinicalTrials.gov search with specified criteria (n = 423)

Records removed before screening:

Duplicate records/compounds removed (n = 74)

**Identification**

Records excluded (n = 234)

Clinical trials withdrawn, suspended, or terminated (n = 72)

Targeted other diagnoses (n = 162)

Records screened (n = 427)

FDALabel (n = 41)

ClinicalTrials.gov (n = 386)

**Screening**

Records assessed for eligibility (n = 193)

Records excluded (n = 160)

Duplicate compound or drug (in RCT) already approved (n = 134)

Over-the-counter supplement or non-drug intervention (n = 12)

Failed to meet primary endpoint or announced to no longer be in development (n = 14)

Compounds included in the review (n = 33)

Approved by FDA (n = 15)

Not approved as of April 2025 (n = 18)

**Included**

Source: Page MJ, et al. BMJ 2021;372:n71. doi: 10.1136/bmj.n71.

This work is licensed under CC BY 4.0. To view a copy of this license, visit <https://creativecommons.org/licenses/by/4.0/>
